# Nutritional, Antioxidant, Antimicrobial, and Toxicological Profile of Two Innovative Types of Vegan, Sugar-Free Chocolate

**DOI:** 10.3390/foods9121844

**Published:** 2020-12-11

**Authors:** Delia Dumbrava, Liviana Alexandra Popescu, Codruța Marinela Soica, Alma Nicolin, Ileana Cocan, Monica Negrea, Ersilia Alexa, Diana Obistioiu, Isidora Radulov, Sofia Popescu, Claudia Watz, Roxana Ghiulai, Alexandra Mioc, Camelia Szuhanek, Cosmin Sinescu, Cristina Dehelean

**Affiliations:** 1 Faculty of Food Engineering, Banat’s University of Agricultural Sciences and Veterinary Medicine “King Michael I of Romania” from Timişoara, Calea Aradului No. 119, 300641 Timişoara, Romania; deliadumbrava@usab-tm.ro (D.D.); ileanacocan@usab-tm.ro (I.C.); monicanegrea@usab-tm.ro (M.N.); ersiliaalexa@usab-tm.ro (E.A.); sofiapopescu@yahoo.com (S.P.); 2 Faculty of Dental Medicine, “Victor Babeş” University of Medicine and Pharmacy, Eftimie Murgu Sq. No. 2, 300041 Timişoara, Romania; livianapopescu@yahoo.com (L.A.P.); cameliaszuhanek@umft.ro (C.S.); minosinescu@yahoo.com (C.S.); 3 Faculty of Pharmacy, “Victor Babeş” University of Medicine and Pharmacy, Eftimie Murgu Sq. No. 2, 300041 Timişoara, Romania; farcas.claudia@umft.ro (C.W.); roxana.ghiulai@umft.ro (R.G.); alexandra.petrus@umft.ro (A.M.); cadehelean@umft.ro (C.D.); 4 Faculty of Agriculture, Banat’s University of Agricultural Sciences and Veterinary Medicine “King Michael I of Romania” from Timișoara, Calea Aradului No. 119, 300641 Timisoara, Romania; almanicolin@usab-tm.ro (A.N.); isidoraradulov@usab-tm.ro (I.R.); 5 Faculty of Veterinary Medicine, Banat’s University of Agricultural Sciences and Veterinary Medicine “King Michael I of Romania” from Timișoara, Calea Aradului No. 119, 300641 Timișoara, Romania; dianaobistioiu@usab-tm.ro

**Keywords:** chocolate, polyphenols, antioxidant activity, antimicrobial activity, toxicological profile, anti-cariogenic activity, sugar-free, vegan

## Abstract

Increased sugar consumption and unhealthy dietary patterns are key drivers of many preventable diseases that result in disability and death worldwide. However, health awareness has increased over the past decades creating a massive on-going demand for new low/non-caloric natural sweeteners that have a high potential and are safer for consumption than artificial ones. The current study aims to investigate the nutritional properties, in vitro toxicological profile, total/individual polyphenols content, and the antioxidant, anti-cariogenic, and antimicrobial activity of two newly obtained vegan and sugar-free chocolate (VHC1 and VHC2). The energy values for the two finished products were very similar, 408.04 kcal/100 g for VHC1 and 404.68 kcal/100 g for VHC2. Both products, VHC1 and VHC2 present strong antioxidant activities, whereas antimicrobial results show an increased activity for VHC1 compared to VHC2, because of a higher phenolic content. In vitro toxicological evaluation revealed that both samples present a safe toxicological profile, while VHC2 increased cellular turnover of dermal cell lines, highlighting its potential use in skin treatments. The current work underlines the potential use of these vegetal mixtures as sugar-free substitutes for conventional products, as nutraceuticals, as well as topic application in skin care due to antimicrobial and antioxidant effects.

## 1. Introduction

In recent decades, consumer demand for food production has changed considerably, and the need for innovative and healthy foods has increased as people became more aware of their role in improving health; this demand is constantly growing worldwide, particularly in developed or developing areas, where the level of information and income are high [1].

Poor dietary habits and a sedentary lifestyle have led to an increase in obesity, diabetes, and other overweight-related diseases among the population, especially in developed countries. Governments, health professionals, and retailers are constantly putting pressure on food producers to reduce the caloric value of processed foods with high sugar content [2]. The necessity to reduce dietary sugar is supported by the high rates of tooth decay when the intake of free sugars exceeds 10% of the total caloric intake, thus justifying the conditional recommendation of 5% free sugars. Evidence shows that sugar plays an essential role in the etiology of tooth decay, with a clear dose-response relationship [3]. Finally, excessive sugar consumption, especially in the form of sugar-sweetened beverages, is also correlated with an increased risk of developing type 2 diabetes and cardiovascular disease [4].

Because of the increase in health awareness, there has been a huge ongoing demand for sugar substitutes that provide fewer or even no calories, and have a better sweetening power than sugar. Thus, a variety of zero-calorie artificial sweeteners have emerged, e.g., saccharin, aspartame, cyclamate, etc. However, artificial sugar substitutes have been associated with health complications therefore their use has subsequently been restricted [5]. Currently, there is a continuous search for low-calorie or non-caloric sweeteners of natural origin, safe for consumption such as Stevia (*Stevia rebaudiana* var. Bertoni), which can be used as a non-nutritive natural sweetener and poses no threat to human health [6].

Cocoa beans and chocolate products have been consumed for thousands of years; for the Maya populations, cocoa beans were a symbol of fertility and life and, according to the legend, chocolate was the “food of Gods.” The Aztecs used cocoa beans as a natural treatment for at least 150 diseases, and during the 17th and 18th centuries, in Europe, chocolate has been used as a medicine to treat various diseases starting from simple colds and cough to digestive diseases, infertility, and even mental disorders [7]. Cocoa is rich in polyphenols with beneficial effects on human health, including especially epicatechin, catechin, and procyanidins [5]. Polyphenols extracted from cocoa are very effective against caries, having a strong antibacterial effect; they cause a significant reduction in the biofilm formation and acid production triggered by *Streptococcus mutans* and *Streptpcpccus sanguinis* [8]. Cocoa has been rediscovered by Western societies more than 3000 years since its discovery, for its benefits in skin treatment due to its complex composition. The bioactive compounds present in cocoa exhibit antioxidant and anti-inflammatory properties based on their ability to inhibit pro-inflammatory cytokines and, subsequently, their downstream metabolic pathways [9]. Among several beneficial systemic effects [10], cocoa powder significantly contributes to skin health, both by oral and topic application, by reversing ageing signs through slowing down the action of free radicals [11]. Because of its anti-inflammatory properties, cocoa powder-based products could be applied on sensitive and dry skin in order to improve the skin texture.

The antimicrobial and antioxidant activities of polyphenols extracts from various plants are being increasingly studied by researchers in the field [12,13,14]. In this regard, carob powder (*Ceratonia siliqua* L. pods), obtained from the fruits of *Ceratonia siliqua*, is frequently used to obtain chocolate-like foods. The carob can be noted for its high content in polyphenols which ensure its antioxidant, antimicrobial, and even anti-tumor effects [15]. Stevia, a species from the Asteraceae family, originating from South America [16], contains two main glycosides: stevioside (5–10%) and rebaudioside A (2–4%). Steviozide is 110–270 times sweeter than sugar whereas rebaudiozide A is 180–400 times sweeter than sugar. These glycosides do not induce a glycemic response when ingested, so Stevia is considered a “noble molecule,” its leaves can be consumed in their natural state as a sweetener, being needed only a small amount and being considered zero calories or low calories. In addition to its natural sweetening properties, as demonstrated by numerous in vitro and in vivo studies, Stevia presents a plethora of biological activities, such as antihypertensive, antioxidant, anti-tumor, anti-carcinogenic, anti-bacterial, anti-inflammatory, anti-diabetic, anti-viral, anti-fungal, and inmunomodulator [17]. Regarding the antibacterial activity of Stevia leaves, it was found that they exhibit significant anti-cariogenic and antibacterial activity on several bacterial strains responsible for the formation and development of dental plaque such as *S. mutans*, *Streptococcus sobrinus*, *Lactobacilus acidophilus* [16].

Green tea (*Camellia sinensis* L.) is known for its high content of polyphenols, especially catechins and for its strong antioxidant activity, having many benefits for human health [18]. Rather recently, researchers have begun to investigate the antimicrobial potential of green tea extracts. It appears that catechins found in green tea present a variety of antimicrobial properties, especially against most oral bacteria [19,20].

Worldwide, cocoa is considered to be a major agricultural product and cocoa-based products are widely used. However, despite the numerous health benefits of cocoa-based products, significant amounts of sugar are added, leading to tooth decay or other severe health problems such as obesity and type 2 diabetes. New natural sweeteners with low calories, similar taste that can also contribute to oral hygiene represents the goal in this particular field of research. This study aims to be an innovative approach in the field of functional foods, by obtaining two vegan, sugar-free hot chocolate, with nutritional, antioxidant, antimicrobial, and anti-cariogenic potential, that can be used as adjuvant and complementary treatment in a healthy diet. Thus, this multipurpose work has taken into consideration the following issues: (i) To obtain a healthy alternative hot chocolate, sweetened with *Stevia rebaudiana* leaf powder instead of sugar and containing, besides cocoa and carob powders, other plants with high polyphenol content and strong anti-cariogenic activity; (ii) to analyze the raw materials as well as the finished products in terms of: nutritional properties, antioxidant activity, and total/individual polyphenols content; (iii) to assess the final product’s antimicrobial activity against nine microbial strains; (iv) to determine the antioxidant activity and nutritional profile of the finished products; (v) to carry out an in vitro toxicological evaluation of these products; and (vi) to analyze the sensorial profile and consumer acceptance of obtained products.

## 2. Materials and Methods

### 2.1. Preparation of the Hot Chocolate Products

Two types of hot chocolate powders were prepared (VHC1 and VHC2), using raw and auxiliary materials purchased from the Romanian market. Both product variants had a common base composition (CBC) obtained by mixing the materials and proportions specified in Table 1. In addition to the common basic composition, other powders obtained from dried vegetable materials were mixed in each type of product (as shown in Table 1), all these being finely ground beforehand with the help of an electric grinder (“Bosch” brand TSM6A013B model, Stuttgart, Germany).

In order to prepare the ready-to-drink hot chocolate, 25 g of chocolate powder mixture from each assortment (VHC1 and VHC2, respectively) was dissolved in 150 mL of hot water (75–80 °C).

### 2.2. Assessment of the Total Polyphenolic Content by the Folin-Ciocâlteu Method

The Folin-Ciocalteu method was employed for the determination of the total polyphenol content in both raw materials and finished products (in both powder and beverage form) [21,22,23]. Briefly, the extract from the powder product samples was obtained after extraction of the 1 g sample with 10 mL ethanol (70%, *v*/*v*) at room temperature for 2 h, stirring occasionally; the resulting alcoholic extracts were filtered and samples were collected for analysis. In the case of ready-to-drink hot chocolate, after filtering the beverage was obtained by dissolving the respective VHC1 or VHC2 powders in hot water, and the samples were taken and analyzed. Total of 0.5 mL of each sample was treated with 1.25 mL Folin-Ciocalteu reagent (Merck, Darmstadt, Germany) diluted 1:10 with water. After the addition of 1 mL Na_2_CO_3_ 60 g/L, (S.C. Chemical Company S.A., Bucharest, Romania), the extract was incubated at 50 °C for 30 min. The absorption of the blue-colored solution was measured at 750 nm using a UV-VIS spectrophotometer (Analytic Jena Specord 205, Jena, Germany). The calibration curve was prepared using gallic acid as standard (concentration range 5–250 µg/mL). All results were expressed as gallic acid equivalents (GAE) per g of sample (mg GAE/g). The experiment was conducted in triplicate and the results were statistically evaluated.

### 2.3. Assessment of Individual Polyphenols by Liquid Chromatography

The identification and quantification of 11 individual polyphenols were conducted by using a Liquid Chromatography-Mass Spectrometer (LC-MS) Shimadzu (Kyoto, Japan) equipment with spectral power distribution ultraviolet lamp SPD-10A UV (Shimadzu, Kyoto, Japan) and LC-MS 2010 (Shimadzu, Kyoto, Japan) detectors and electro chemical (EC 150/2 NUCLEODUR C18 Gravity SB 150 × 2 mm × 5 um) separation columns. Gradient mobile phases were used for the separation of compounds: water with formic acid at pH-3 (mobile phase A), acetonitrile with formic acid at pH-3 (mobile phase B), gradient program: 0.01–20 min 5% B, 20.01–50 min 5–40% B, 5–55 min 40–95% B, 55–60 min 95% B, mobile phase 0.2 mL/min, temperature 20 °C. The monitoring wavelengths were 280 nm and 320 nm and the calibration curves were built between 20 and 50 µg/mL. The experiment was conducted in duplicate and results were expressed in mg/g.

### 2.4. Assessment of the Antioxidant Capacity (AC) by Cupric Reducing Antioxidant Capacity Assay (CUPRAC)

The Cupric reducing antioxidant capacity (CUPRAC) method [24] was used in order to establish the antioxidant activity (AC) of the final products. Briefly, 1 mL CuCl_2_ 0.01 M solution (S.C. Chemical Company S.A., Bucuresti, Romania), was mixed with 1 mL neocuproine (7.5 × 10^−3^ M) (2,9-Dimethyl-1,10-phenanthroline) and 1 mL acetate buffer (Merck, Darmstadt, Germany). The resulting solution was mixed with 1.1 mL alcoholic extract (preparation see Section 3.2) and incubated for 30 min at 20 °C. The solution absorption was measured at 450 nm using an ultraviolet–visible (UV-VIS) spectrophotometer (Analytic Jena Specord 205, Jena, Germany); Trolox (6-hydroxy-2,5,7,8-tetramethilcroman-2-carboxylic acid) (Merck, Darmstadt, Germany) was used as the reference. All experiments were carried out in triplicate.

### 2.5. Assessment of Proximate Composition, Nutritional Profile, and Energy Value

The proximate composition of the finished products (VHC1, VHC2) was assessed according to ISO Methods: moisture SR ISO 1442:2010, protein SR ISO 937:2007; total lipid SR ISO 1443:2008; mineral substances SR ISO 936:2009, sugar SR ISO 91-2007, and fiber according ISO 13906: 2007; with FOOS Fibertec 2010&M6, Sweden. The carbohydrate content (%) was calculated as the difference between 100 and the sum of the following fractions: lipids, proteins, ash, fiber, and moisture.

The caloric intake, also known as the energy value, is calculated by summing the caloric intake produced by individual nutrients (lipids, carbohydrates, and proteins) by taking into account the following correlations: 1 g lipids = 9 kcal, 1 g protein = 4 kcal, 1 g carbohydrates = 4 kcal.

### 2.6. Assessment of Antimicrobial Activity

The two chocolate preparations, VHC1 and VHC2, were tested for their antimicrobial activity on bacterial and fungal strains obtained from our own laboratory culture collection (Laboratory of Microbiology, Interdisciplinary Research Platform within Banat’s “King Michael I of Romania” University of Agricultural Science and Veterinary Medicine Timisoara). The bacterial strains used are as follows: *Staphylococcus aureus* (ATCC 25923), *S. mutans* (ATCC 19615), *Escherichia coli* (ATCC 25922), *Pseudomonas aeruginosa* (ATCC 27853), *Shigella flexneri* (ATCC 12022), *Salmonella typhimurium* (ATCC 14028), and *Haemophillus influenzae* type B (ATCC 10211) whereas the fungal strains used are *Candida albicans* (ATCC 10231) and *Candida parapsilopsis* (ATCC 22019). The strains were maintained at −50 °C and were revived prior to the experiment day by overnight incubation at 37 °C in brain heart infusion (BHI) broth (Oxoid, CM1135).

In order to assess the antimicrobial activity, the microbial mass loss was evaluated by means of optical density (OD) measurement using an ELISA microplate reader (BIORAD PR 1100, Tallin, Estonia), in accordance with ISO 20776-1:2019 procedure. The OD of the VHC1 and VHC2 samples was recorded at 540 nm.

For each bacterial and fungal strain tested, a fresh culture 10^−3^ fold dilution, presenting an inoculum equivalent to 0.5 McFarland standard (1.5 × 10^8^ CFU/mL), was used to perform the analysis. The resulting suspensions (100 μL) were added to a 96-well flat-bottomed microdilution plate (total volume of 200 μL). Increasing volumes (25, 50, 75, and 100 µL) of ready-to-drink products (VHC1 and VHC2 beverages) were applied on each bacterial and fungal suspension used [25]. The OD was measured in the initial moment, thus obtaining the ODT0 values. The plates were covered and left for 24 h incubation at 37 °C, followed up by the OD measurement (ODT24). The tests were performed in triplicate for all samples; the mean values were calculated and used subsequently.

The simple strains suspensions in BHI were used as a positive control; the results were calculated using the following formula (1):

ODanalysed sample = ODT24 − ODT0(1)

where:

ODT0—optical density at T0 (nm)

ODT24—optical density at T24 (nm)

### 2.7. In Vitro Toxicity Assay

The two chocolate products (VHC1 and VHC2) were subjected to in vitro toxicological tests, using healthy human cell lines (primary gingival keratinocytes, human skin keratinocytes—HaCat and human skin fibroblasts—1BR3). Human primary gingival keratinocytes cell line (ATCC^®^ PCS-200-014 ™) was purchased from ATCC (American Type Cell Collection), HaCat—immortalized human keratinocytes was purchased from CLS Cell Lines Service GmbH (catalog no.: 300493), while the human fibroblast cell line 1BR3 was acquired from ECACC (European Collection of Authenticated Cell Cultures, catalog no.: 90011801), in the form of frozen vials. The culture media and growth kits were purchased as follows: Dermal Cell Basal Medium (ATCC^®^ PCS-200-030 ™) and Keratinocyte Growth Kit (ATCC^®^ PCS-200-040 ™) from ATCC, Dulbecco’s modified Eagle medium (DMEM) with a high glucose content of 4.5 g/L, EMEM (Eagle’s minimum essential medium) as well as other necessary reagents for cell culture such as phosphate saline buffer (PBS), trypsin/EDTA solution, FBS (fetal bovine serum), penicillin/streptomycin solution and Trypan blue from Sigma Aldrich (St. Louis, MO, USA).

#### 2.7.1. Cultivation of Cell Lines

The cells were stored in liquid nitrogen until thawed and cultured; cells were cultured in culture-specific medium—Dermal Cell Basal Medium (gingival keratinocytes) in which a specific growth kit was added—Keratinocyte Growth Kit, Dulbecco’s Modified Eagle’s Medium (DMEM) supplemented with 10% Fetal Bovine Serum (FBS) (HaCat cells) and Minimum Essential Medium Eagle (EMEM) supplemented with 15% FBS (1BR3 cells) and kept in a humid atmosphere in the incubator at 37 °C and 5% CO_2_. The cells were split every 2–3 days, and then counted in the presence of Trypan blue by using the Countess II FL Automated Cell Counter, Thermo Fisher Scientific, Waltham, MA, USA.

#### 2.7.2. Alamar Blue Assay for Cellular Viability

In order to assess the potential cytotoxic effects of the two products (VHC1 and VHC2) against the above-mentioned cell lines, the cells were cultured in 96-well plates (10^4^ cells/well) and allowed to adhere to the plate until reaching a suitable confluence (24–48 h). Stock solutions were prepared in culture medium (10 mg compound/mL) and the following concentrations were tested for each recipe: 5, 10, 25, and 50 µg/mL (cocoa was used as reference) for 72 h. After 72 h, 20 µL Alamar blue was added and the plates were incubated for 3 h at 37 °C; the absorbance values were read at 570 nm and 600 nm, respectively, by using the xMark Microplate Spectrophotometer, Biorad, 168–1150, Hercules, CA, USA).

The following formula (2) was used in order to calculate the cell viability [26]:

{[(εOX)λ2 Aλ1 − (εOX)λ1 Aλ2 of test agent dilution]/[(εOX)λ2 A°λ1 − (εOX)λ1 A°λ2 of untreated positive growth control]} × 100(2)

where:

εOX = molar extinction coefficient of Alamar Blue oxidized form (BLUE);

A = absorbance of test wells;

A° = absorbance of positive growth control well (cells without tested compounds);

λ1 = 570 nm and

λ2 = 600 nm

### 2.8. Sensory Evaluation

Sensory evaluation (consumer preferences) of the VHC1 (chocolate with plant extract) and VHC2 (chocolate with dehydrated fruits powder) was carried out by ten untrained panelists (4 males and 6 females) with ages between 22 and 50, non-smokers, regular chocolate consumer, without known cases of food allergies. The VHC 1 and VHC 2 samples were presented to panelists in 100 mL transparent glass labeled with four-digit characters, in a single sensory session. The panelists were asked to evaluate the following sample attributes: color, consistency, flavor, taste, and overall acceptability. For this assessment a five-point hedonic scale was used [27], as follows: 1 = extremely dislike; 2 = slightly dislike; 3 = neither like nor dislike; 4 = slightly like; 5 = extremely like.

The level of acceptability and ranges of score were classified as follows: 4.5–5.00 = highly acceptable (HA); 3.5–4.49 = acceptable (A); 2.50–3.49 = moderately acceptable (MA); 1.5–2.49 = slightly acceptable (SA); 1.00–1.49 = not acceptable (NA). Panelists were asked to rinse their mouth with still water in between sample evaluation.

### 2.9. Statistical Analysis

For polyphenols content, antioxidant activity, nutritional values, microbiological assay, and in vitro toxicological assay, the mean values and standard deviations of all replicates were calculated using Excel software (Microsoft Office 2010, MSO) and GraphPad Prism (v.5.0 software, Manufacture, San Diego, CA, USA). Differences between means were analyzed with a one-way ANOVA, followed by multiple comparison analysis using the T test (two-sample assuming equal variances) and Tukey’s post-hoc test. Differences were considered significant when *p*-values < 0.05.

## 3. Results and Discussion

### 3.1. Total Polyphenols (TP) and Individual Polyphenols Content

The results regarding the TP content of raw materials and final products are depicted in Table 2 expressed as gallic acid equivalents (GAE). One can notice that the highest content of TP can be found in cloves (*Syzygium aromaticum* L.) powder (130.9 ± 2.71 mg GAE/g) and cocoa (109.70 ± 1.93 mg GAE/g), while the lowest value was obtained in soy lecithin (11.04 ± 0.45 mg GAE/g).

The final products VHC1 and VHC2 obtained as powders exhibit high values of polyphenol content (127.81 ± 2.33 and 137.7 ± 2.23 mg GAE/g, respectively). In the ready-to-drink products based on the use of VHC1 and VHC2 powders, the TP content is lower (17.14 ± 0.55 mg GAE/g and 18.50 ± 0.52 mg GAE/g). The difference occurs because of the technological process of beverage preparation that uses water as solvent which has weaker polyphenol extraction capacity than ethanol. As presented above, cocoa powder is one of the richest matrix in TP, previous studies reporting similar results [28,29]. Regarding alfalfa (*Medicago sativa* L.), studies have found that the TP content depends on the agro-climatic conditions [30] and part of the plant used; the highest values are found in leaves extract [31]. Moreover, the level of TP can be increased by means of ultrasonic-assisted extraction, as reported by Jing et al. [32].

Green tea is a matrix recognized for its antioxidant properties and high content in polyphenols, particularly tannins. Similar TP content values as the ones recorded in the current study (>100 mg GAE/g) have been reported by most researchers in alcoholic or aqueous extracts of green tea [33,34,35].

The plants belonging to the *Lamiaceae* family are very reach in TP [36]; sage (*Salvia officinalis* L.) was widely studied for its therapeutic properties generated by its high polyphenol concentration. In the current study we found a value of 59.14 ± 1.47 mg GAE/g in the sage ethanolic extract which falls within the values reported by several other researchers [37,38]; however higher TP values have been reported in the ethanolic extract obtained from the aerial parts of sage collected from the spontaneous Romanian flora [36]. Our extraction method yielded rich-TP carob extracts (Table 2); the results are in line with other studies that shows the benefit of using aqueous-polar solvents for high-TP content extraction [39]. In the blueberry (*Vaccinium myrtillus* L.) samples we recorded a TP concentration of 47.09 ± 1.52 mg GAE/g, value which falls within the literature reported range [40]. Moreover, a dependency of TP concentration on the plant variety and the agro-climatic conditions was reported in blueberry [41]. In blackcurrant the literature reports lower TP values as compared to those recorded in the current study [42].

In clove extracts, studies highlight yet again the influence of the solvent on the TP content [43,44]; our recorded value (130.9 ± 2.71 mg GAE/g) stands at the upper limit of the range of concentrations mentioned in the literature. The lowest intake of TP is given by soy lecithin (11.04 ± 0.45); low TP values for soy lecithin were reported by another group of researchers [45].

Stevia plays an important role in the preparation of hypoglycemic products; given its low glycemic index, stevia also contributes to a significant intake of total polyphenols. The TP content of our ethanolic extract (103.36 ± 2.74 mg GAE/g) was higher than the values reported by other authors [46,47,48,49] and by contrast, lower than the values reported by the group of Ana et al. [50].

It is worth noting that the values found in terms of total and individual polyphenols in plant materials depend not only on the pedoclimatic conditions but also on the plant drying method as well as the solvent used in the polyphenol extraction process.

In terms of individual polyphenols, phenolic acids were determined such as: gallic acid (GA), protocatechuic acid (PR), caffeic acid (CA), coumaric acid (CU), ferulic acid (FE), and rosmarinic acid (RO); we also determined the content in several flavonoids, such as: epicatechin (EC), rutin (RU), resveratrol (RS), quercitin (QU) and kaempherol (KP); the values of polyphenolic content varied according to the analyzed matrix (Table 3).

Gallic acid (GA) content in plant materials used in our recipes ranges between 0.14 ± 0.007 mg/g in black currant (*Ribes nigrum* L.) powder and 23.98 ± 0.764 mg/g in carob powder (Table 3). Analyzing the literature available we found that we obtained a higher GA concentration in carob pods and lower GA in black currant and cacao powder, than those reported by other studies [51,52,53,54,55]. In the alfalfa extract we obtained similar results to those reported by Karimi et al. [31]. Interestingly, our investigation was not able to identify the presence of GA in sage, as compared to a previous study [56].

Compared to recent literature data, our study identified lower protocatechuic acid (PR) content in carob, cacao, clove, vanilla, black currant and cardamom (*Eletaria cardamomum*, L.) [54]. Contrary to other studies, in stevia we were not able to identify the presence of PR [53].

Caffeic acid (CA) was the only hydroxycinamic acid identified in all analyzed matrices. CA values obtained in our study are similar with those of Irondi et al. [55] and higher as compared to the values obtained by the group of Gaweł-Bęben et al. [57].

Similar values to those reported by literature data were observed for the coumaric acid (CU) in blackcurrant [53] and sage [56].

The highest ferulic acid (FE) content was identified in the green tea extract whereas the lowest values were detected in sage, as previously reported by other studies [56]; however, the literature reports much higher concentrations of FE in stevia (0.86–5.5 mg/g) compared to our experimental data [57].

In stevia, when analyzing the results, we observed that rosmarinic acid (RO) concentration is higher than those presented in the literature [57]; however, similar concentrations were found for epicatechin (EC) [57] and lower concentration for quercetin (QU) [58].

In addition to the hydroxycinamic acids, we investigated the amount of QU and kaemferol (KE) flavonoids, polyphenolic compounds found in many fruits and vegetables [59]. QU has a bitter flavor and is used as an ingredient in dietary supplements, beverages, and foods. As expected, the medicinal plants of the Lamiaceae family (sage and thyme) as well as clove are characterized by a high content of flavonoids [60]; however, we obtained the highest values of QU and KE in stevia (4.87 ± 0.09 mg/g QU and 4.36 ± 0.085 KE).

EC is a monomeric flavanol present in noteworthy concentrations in cocoa powder, chocolate, and tea [61]; as presented in Table 3, except for cardamom and lecithin, all other assessed matrices exhibit EC in their composition. The highest EC values were detected in vanilla while the lowest value of EC was detected in stevia. Similar EC results were reported in literature studies for cocoa powder [62] and green tea [63].

As mentioned above, because of the extractive solvent used for the analysis of individual polyphenols (ethanol for VHC1/VHC2 powder and hot water for ready-to-drink VHC1/VHC2 beverages), there is a significant difference between the TP content of the two finished products. Also, as expected, the amount of all individual polyphenols was significantly higher in the powdered products compared to the ready-to-drink beverages. However, kaempherol (KP) was the only exception; its concentration was higher in VHC1 ready-to-drink product than in the VHC1 powder product.

**Figure 1:**
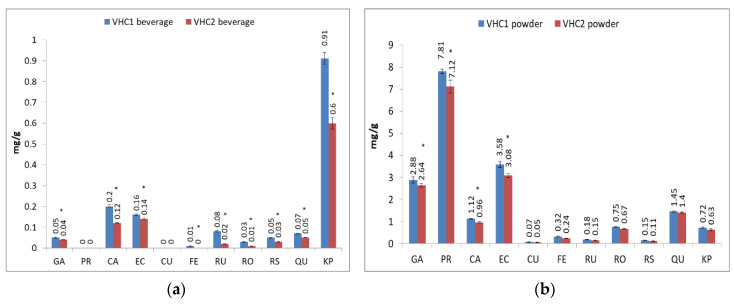
Individual polyphenols in final products vegan hot chocolate 1 (VHC1) and vegan hot chocolate 2 (VHC2) as beverages (**a**) and powders (**b**) (mg/g), respectively. * superscript between final products indicate significant differences (*p* < 0.05) between values according to the T test.

Comparing them, in both ready-to-drink and powder form, the VHC1 variant had slightly higher individual polyphenol content than the VHC2 variant. Protocatechuic acid (PR) was best represented in the matrices of the powdered products (7.81 mg/g in VHC1 and 7.12 mg/g in VHC2), it was not identified in the matrices of ready-to-drink products (Figure 1). Coumaric acid (CU) was also not identified in ready-to-drink products, but it was present in relatively small quantities (0.07 mg/g in VHC1 powder and 0.05 mg/g in VHC2 powder) in powdery ones. Ferulic acid (FE) was present in the matrices of the powder products (0.32 mg/g in VHC1 powder and 0.24 mg/g in VHC2 powder) but it was identified only in the ready-to-drink VHC1 (0.01 mg/g) product. In the matrices of ready-to-drink products, the best represented after KP was CA (0.20 mg/g) and EC (0.16 mg/g) for VHC1, as well as EC (0.14 mg/g) and CA (0.12 mg/g) for VHC2 (Figure 1).

### 3.2. Antimicrobial Activity

**Figure 2:**
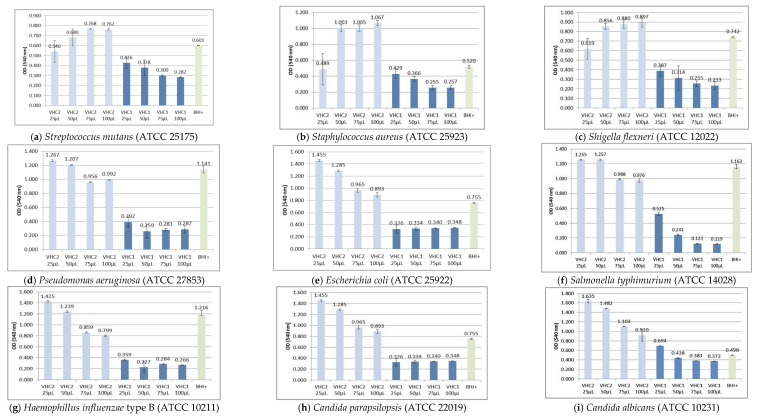
Antimicrobial activity expressed as OD (mean ± SD) values obtained according the formula (1). BHI+: simple strains suspensions in brain heart infusion broth used as a positive control.

Figure 2 exhibits the antimicrobial activity of VHC1 and VHC2 samples on the nine tested bacterial strains; one can notice that on all strains the VHC1 product induces lower optical density (OD) values compared to VHC2 thus indicating higher antimicrobial activity. For the VHC1 product, lower OD values compared to the reference sample were recorded for all tested strains and concentrations, respectively, which denote an antibacterial activity exerted through the inhibition of the micellar development. VHC2 exhibited higher than reference OD values for most strains and concentrations. For *S. mutans, S. aureus,* and *S. flexneri* the OD values increase with increasing VHC2 concentrations thus emphasizing the stimulatory effects on the micellar development induced by the main component matrices. On the *E. coli*, *P. aeruginosa*, *S. typhimurium*, and *H. influenzae* type B cultures, VHC2 produced weak antimicrobial effects when 75 si 100 µL were applied, as reflected by its lower OD values compared to the reference; for the fungal cultures of *C. albicans* and *C. parapsilopsis*, increased product concentrations lead to the inhibition of micellar development but the OD values are superior to the control brain heart infusion (BHI) sample. Considering the composition of the two analyzed products, VHC1 and VHC2, and their effects on the tested bacterial cultures, one can conclude that the VHC1 antibacterial effects are attributable to its components (cardamom, wild thyme—*Thymus serpyllum* L., green tea, alfalfa, sage, Echinacea—*Echinacea purpurea* L.) while the VHC2 components (black currant, blueberry, hibiscus—*Hibiscus rosa-sinensis* L. and clove) exhibit weaker antimicrobial effects.

Plants produce numerous secondary metabolites with antimicrobial properties as part of their normal growth process with the purpose to inhibit the attack of environmental pathogens; therefore, plant extracts are usually revealed with effective antibacterial properties. Previous studies have identified antibacterial and antifungal effects for: (i) black currant (*E. coli*, *A. niger, P. vulgaris, C. albicans*—[64]); (ii) clove and thyme (*S. aureus, P. aeruginosa, E. coli, S. pyogenes, Corynebacterium* spp, *Salmonella* spp, *B. fragilis, C. albicans*—[65]); (iii) blueberry (*L. monocytogenes, S. enteritidis*—[66]); (iv) hibiscus (*S. typhimurium, S. aureus*—[67]); (v) green tea (*S. mutans*, dental bacteria—[68]); (vi) cardamom (*C. albicans, S. mutans, S. aureus, L. monocytogenes, B. cereus, S. typhimurium*—[69]); (vii) alfalfa (*S. pneumonia, H. influenza, M. catarrhalis*—[70]); (viii) sage (*S. mutans, S. aureus, E. coli*—[71]); (ix) Echinacea (*Burkholderia cepacia* complex bacteria—[72]). One mechanism through which plant extracts exhibit antimicrobial properties is the synergism between phytochemical compounds or between phytochemicals and associated antibiotics [73]; the synergistic mechanism can be explained by the association of numerous different molecules in plant extracts so that bacterial resistance through genetic mutations triggered by external stimuli is more difficult to develop [74]. In addition, the presence of inactive compounds in plant extracts may influence the absorption, metabolization rate, and subsequently, the bioavailability of active compounds [75]. In our study, the association of various plant extracts in the VHC1 product produced a strong antibacterial effect as a result of the combined activities of individual extracts and active compounds, which may act in an additive or synergistic manner.

However, in certain cases, an antagonistic effect can be recorded when multiple natural compounds are associated; this behavior can be attributed to the presence of inactive compounds in plant extracts that are able to act as growth substrate for bacteria [75]. Nevertheless, antagonistic effects occur much less frequently than synergistic ones, contrary to the older beliefs [76]. In our study, the antibacterial activity of VHC2 product was much weaker compared to VHC1 despite the association of plant extracts with previously documented antimicrobial properties; we can hypothesize that the VHC2 poor antibacterial effect is caused by the antagonistic reactions between components. The antibacterial activities of the final products (powders and beverages), higher in VHC1 than in VHC2, cannot be correlated with their total polyphenolic content; polyphenols have been reported by many sources as antioxidant as well as antibacterial and antifungal active compounds but so far, the scientists have not reached a clear conclusion whether these two effects are related or not. However, numerous plant extracts have exhibited both antioxidant and antibacterial properties because of their high polyphenolic content [77] which largely depends on the extraction process. For our two final products the main difference consists in their individual flavonoid percentages; therefore we might assume that the VHC1 product owes its antibacterial effect to the presence of flavonoids which act through the formation of complexes with bacteria and the inhibition of energy metabolism and DNA synthesis [25]. In addition, the presence of gallic acid in the product composition promotes the antibacterial activity of EC by inducing damages to the bacterial membrane [25]; however, if we compare Gram-positive to Gram-negative bacteria, one can notice that the antibacterial activity of final products is not specific for either class but rather strain-dependent, the strongest antibacterial effect being exerted on *S. typhimurium*. The weakest antibacterial effects were noticed on *E. coli* and *S. mutans strains;* for E. coli strain, this effect may be explained by the presence of an extra outer membrane which may hamper the membrane permeability to the plant extract [78,79]. Contrary to our results, on other strains of *S. mutans* polyphenols exhibited a strong antimicrobial effect by decreasing the bacterial growth and changing their architecture [80]. Therefore, we may conclude that the antimicrobial activity strongly depends on the specific strain used.

### 3.3. Antioxidant Activity Assessed by CUPRAC Method

The antioxidant activity of the finished beverage products was determined by using the CUPRAC method (Table 4), both products showing strong antioxidant activities, with 38,608 mg Trolox/g obtained for VHC1, a slightly higher value than the one found for VHC2 (37,258 mg Trolox/g).

The CUPRAC method was introduced in 2004 by Apak et al. as a total antioxidant capacity assay in order to provide some advantages to other previously applied methods; thus the copper(II)-neocuproine (Cu(II)-Nc) reagent is easily affordable, soluble in both aqueous and organic media, stable and selective, provides faster kinetics, can be used at physiological pH and responds to all types of antioxidants including the ones that can be found in foods and beverages [24,81]. Apak et al. estimated in a comprehensive review published in 2007 that the CUPRAC method meets six out of the eight criteria proposed to define an ideal standardized antioxidant capacity assay as follows: simplicity, clear end-point and mechanism, readily available equipment, intra- and inter-assay reproducibility, ability to simultaneously respond to hydrophilic/hydrophobic antioxidants, high throughput for routine analysis [82].

The antioxidant capacity of plant extracts is an important parameter in their biological assessment because of the fact that high antioxidant properties increase the shelf life of the final product as well as its flavor without the need to use synthetic compounds with carcinogenic and/or mutagenic potential [83]. Our study indicated significant antioxidant effects for both hot chocolate types, in agreement with their polyphenolic content.

### 3.4. Nutritional Profile of the Finished Products

The proximate composition of the two powder products is exhibited in Table 5.

One can notice that both products exhibit very close values in terms of protein, lipid, and carbohydrate content. Also, carbohydrates predominate in both VHC1 and VHC2 chocolate assortments (69.36 g/100 g and 71.63 g/100 g, respectively), followed by proteins (11.11 g/100 g and 11.45 g/100 g, respectively); among carbohydrates, dietary fibers (28.37 g/100 g and 29.30 g/100 g, respectively) are in higher concentration than sugars (15.35 g/100 g and 15.85 g/100 g, respectively) and the latter come only from the vegetable raw materials used in the extraction process. The energy values for the two finished products are also very similar, 408.04 kcal/100 g for VHC1 and 404.68 kcal/100 g for VHC2.

### 3.5. In Vitro Toxicological Evaluation of Finished Products

**Figure 3:**
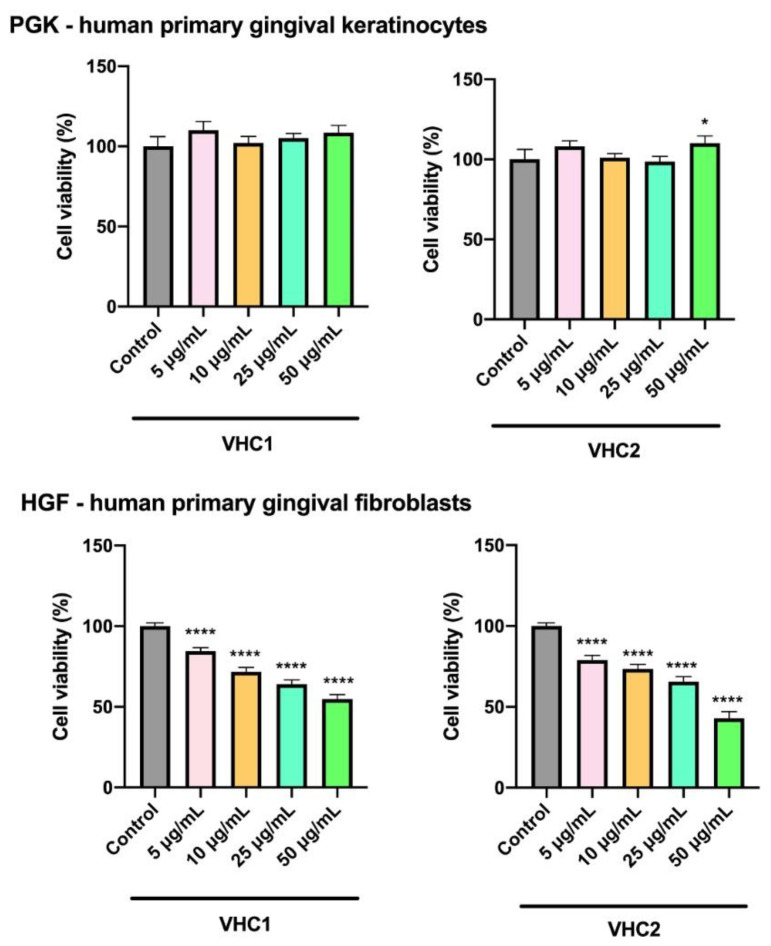
The effect of VHC1 and VHC2 on the viability of human gingival cell lines—primary gingival keratinocytes (PGK) and human primary gingival fibroblasts (HGF) at 72 h post-stimulation. The cell viability percentage was normalized to control cells (no stimulation), considered 100% cell viability. The data represent the mean values ± SD of three independent experiments. One-way ANOVA analysis was applied to determine the statistical differences followed by Tukey’s multiple comparisons test (* *p* < 0.05; **** *p* < 0.0001).

The in vitro toxicological evaluation of the finished products was carried out on primary gingival keratinocytes by the assessment of cell viability through the Alamar Blue test. The primary gingival keratinocytes are normal gingival epithelial cells with similar properties as the dermal epithelial cells and are frequently used for the analysis of the oral epithelium properties [84]. The experimental data obtained for the VHC1 indicated that after 72 h stimulation all tested concentrations (5, 10, 25, and 50 µg/mL, respectively) did not affect cell viability; moreover, they induced a slight stimulation of their number. Similar results were recorded for the VHC2 product (Figure 3).

However, the viability of human primary gingival fibroblasts—HGF cells decreases with increasing concentration of VHC1 and VHC2 samples; the application of up to 10 µg/mL VHC1 and VHC2, respectively, on HGF cells induces an acceptable viability level of over 70%. In contrast, HGF cells stimulated with concentrations of 25 µg/mL of each product, respectively, exhibited 64% cell viability for VHC1 and 65.63% cell viability for VHC2 sample. The highest applied concentration (50 µg/mL) induced a decrease in HGF cell viability, quantified at 54.81% for VHC1 and 42.97% for VHC2 (Figure 3).

**Figure 4:**
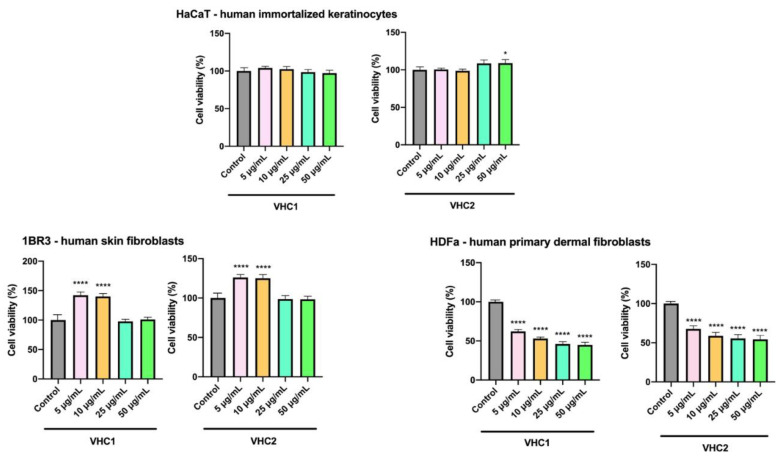
The effect of VHC1 and VHC2 on the viability of human skin cell lines—human immortalized keratinocytes—HaCaT, human skin fibroblasts—1BR3, and human primary dermal fibroblasts—HDFa, at 72-h post-stimulation. The cell viability percentage was normalized to control cells (no stimulation), considered 100% viability. The data represent the mean values ± SD of three independent experiments. One-way ANOVA analysis was applied to determine the statistical differences followed by Tukey’s multiple comparisons test (* *p* < 0.05; **** *p* < 0.0001).

The effect of the two chocolate products VHC1 and VHC2 was also tested on three healthy skin cell lines, human immortalized keratinocytes—HaCaT, human skin fibroblasts—1BR3, and human primary dermal fibroblasts—HDFa (Figure 4). One can notice that no toxicity effects occurred on the healthy HaCaT and 1BR3 skin cells after the application of both products even for the highest tested concentrations (50 µg/mL); moreover, both products induced a stimulatory effect, especially on 1BR3 fibroblast proliferation rate when applied in low concentrations (5 and 10 µg/mL, respectively) (Figure 4). These data indicate the lack of cytotoxic effects for the two tested products VHC1 and VHC2 on the respective human skin cell lines.

However, HDFa cell viability (Figure 4) follows the same trend as HGF cells (Figure 3), where low concentrations (5 and 10 µg/mL) exhibit less harmful effect; still, the sensitivity of HDFa cells to the tested samples is higher compared to the one of HGFa cells. HDFa cells treated with the VHC1 sample exhibit a viability percentage between 62.13% and 44.95% depending on concentration, while the cell population treated with VHC2 sample seems to be less affected than the HDFa cells stimulated with VH1C sample, the cells showing a viability between 67.63% and 54.32% vary in a concentration-dependent manner (Figure 4).

The assessment of biocompatibility and cytotoxicity is nowadays a mandatory part of the initial evaluation of medical devices and therapeutic compounds as stipulated by ISO standards. Additionally, in vitro toxicological studies are essential in the screening process of any product intended for human consumption, before market approval [85]. Currently, cell cultures originating from human oral tissue have many applications in the evaluation of oral products, including effects of drugs or other bioactive compounds [86]. The selection of the cell types used in our work was made based on the fact that gingival keratinocytes and fibroblasts are among the most abundant resident cells in the oral mucosa which will have a direct contact with our proposed products. Human gingival fibroblasts exhibit a higher ability to induce scarless wound healing compared to human skin fibroblasts, because of the different release of growth factors [87].

In addition, healthy skin keratinocytes and fibroblasts were used as well for the in vitro testing of the products in order to: (1) Establish a comparison between oral and dermal cells in terms of sensitivity to the tested products, and (2) assess a potential future use of the products in skin care treatments for cosmetic use. The in vitro testing of cosmeceuticals on cell lines represents a valuable tool in the assessment of skin damage, providing reproducible results under controllable test conditions and an alternative to animal use [88].

Our in vitro studies involved primary gingival and dermal keratinocytes and fibroblasts as well as immortalized skin cell lines; studies indicate that primary cell cultures may provide more useful clinical data than immortalized ones due to their natural ploidy and similar biological parameters with in vivo cells [87]. Immortalized cells exhibit genotype and/or phenotype alterations that may result in different reactions to external stimuli; therefore, the use of primary cells is recommended in order to better mimic in vivo testing [88]. Also, primary human oral mucosal cells are superior to animal-related cell lines in terms of toxicity screening [87].

Our study on human gingival keratinocytes as well as on the two healthy skin cell lines (human keratinocytes, HaCaT, and human fibroblasts, 1BR3 revealed not only a lack of cytotoxicity for both products but stimulatory effects in terms of cell growth. In a normal environment, healthy mammalian cells divide and multiply; the presence of a toxic agent causes the decrease of cell viability [11]. The addition of either product, VHC1 or VHC2, respectively, in various concentrations, to healthy cells has resulted in two distinct events: (1) The product did not affect cell viability, thus indicating the suitability of the product for safe administration in humans, without adverse effects; and (2) the product increased cell growth thus indicating cell turnover and resulting in the formation of new cells [11]. When the two final products were applied on human gingival keratinocytes one could notice that all tested concentrations induced a slight stimulatory effect on cell viability. This result suggests that both products are safe for oral administration and more importantly, lays the groundwork for further studies that will prove that the tested products are beneficial for different oral mucosa pathologies. The two dermal cell lines reacted differently to the two products; the HaCaT cells showed no cytotoxic effects after the application of the VHC1 product, regardless of concentration, while cell turnover occurred when the VHC2 product was applied in high concentrations. This behavior recommends the VHC2 product for topical application against skin ageing as it provides the formation of new skin cells thus making the skin more smooth and supple; the VHC1 product, although it does not provide stimulatory effects on the skin cells, is considered nontoxic and can be used safely in the biomedical field. Dermal fibroblasts 1BR3 showed a similar reaction to both chocolate products: low concentrations of the product induced cell growth while high concentrations were documented as nontoxic; therefore, we may conclude that the two chocolate products can be safely applied on human skin even in high concentrations but low concentrations should be used in skin treatments.

The situation changes when the final products are tested on primary dermal and gingival fibroblasts, respectively, when cytotoxic effects were recorded in a dose-dependent manner, dermal cells being more sensitive compared to gingival ones. Similar results were reported by Bullock et al. in 2020 who revealed that oral fibroblasts were more sensitive to external agents than oral keratinocytes [89]. We may assume that because of stem-like features and higher proliferation rates keratinocytes exhibit superior resistance to external agents compared to fibroblasts.

Collectively, these data reveal the suitability of the final products to be used occasionally as beverages. Moreover, considering the fact that VCH1 increases the cellular viability only in 1BR3 human fibroblasts, whereas VHC2 increases cellular turnover in both tested cell lines (1BR3 and human keratinocytes HaCaT), one can assume that the VHC2 chocolate can be safely administered as topic treatment in skin anti-ageing treatments. The superior effects of VHC2 at skin level could be attributed to the black currant and blueberry components which were previously recommended as the natural antioxidant best choice for foods and health products [90].

### 3.6. Sensory Analysis

In order to evaluate the consumer’s acceptability regarding the consumption of the hot chocolate products, they were sensorially tested by a group of men and women panelists. The male panelists ranged in age from 20 to 50 and the women in the 20–41 age group.

**Figure 5:**
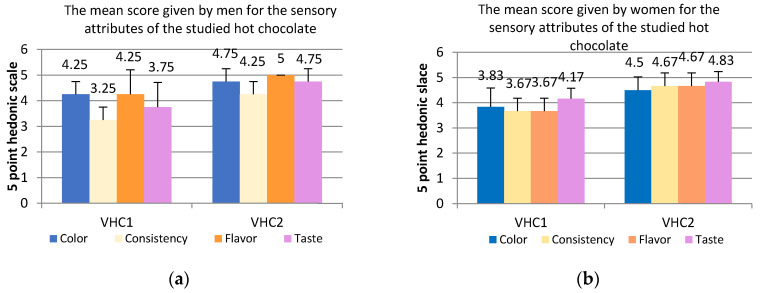
Mean scores of sensory attributes (color; consistency; flavor and taste) for chocolate samples (VHC1 and VHC2) depending on gender ((**a**). male; (**b**). women).

Figure 5 indicates the average score values given by female and male panelists to the chocolate studied samples VHC1 and VHC2; it can be clearly noted that men gave the maximum score of 5 points to the VHC2 chocolate sample, for flavor attribute, followed by color (4.75) and taste (4.75), using a 5-point hedonic scale. Less appreciated by men was the consistency and taste of the VHC1 chocolate sample which obtained 3.25 and 3.75 respectively (Figure 5a).

Women rated the VHC2 sample with highest score for taste (4.83 points), followed by flavor and taste attributes with the same score 4.67 (Figure 5b). The VHC1 chocolate sample was less appreciated by women, compared to the HCV2 sample obtaining for the taste and color attributes 4.17 and 3.83 respectively. The lowest scores were given to the consistency and flavors obtaining 3.67 points each (Figure 5b).

**Figure 6:**
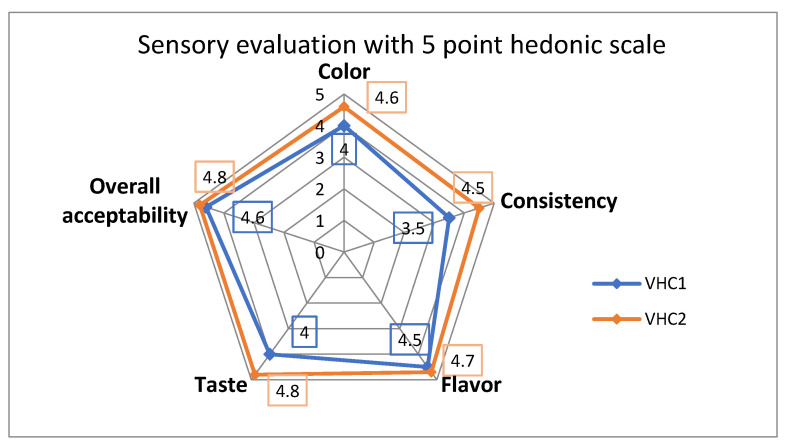
Global values of the sensory evaluation of VHC1 and VHC2 by using a 5-point hedonic scale.

The mean scores (men and women) for the sensory attributes of the studied chocolates prepared from mixtures of medicinal plants and fruits are shown in Figure 6. The highest appreciated was the VHC2 sample which obtained a mean score of 4.8 for overall acceptability, 4.6 for color, 4.5 for consistency, 4.7 for flavor and 4.8 for taste, thus falling within the 4.5–5.00 score range which indicates high acceptability (HA). The VHC1 test falls within the 3.5–4.49 level of acceptability (acceptable—A) for consistency, color and taste; in terms of aroma and overall acceptability, it falls into the highly acceptable class (HA) (Figure 6). Similar studies regarding sensory evaluation of chocolate have been carried out by Iserliyska et al. in 2007 and Norhayati and Mohd in 2014 [91,92].

The results of the sensory evaluation, which highlights the consumers’ preferences, underline the fact that the samples of chocolate with dehydrated fruit powder (VHC2) were highly appreciated by the evaluators, obtaining a very high score, thus being able to classify this sample in the highly acceptable quality class (HA).

To date, our studies on the two innovative varieties of hot sugar-free chocolate have focused on the analysis of total and individual polyphenols content, antioxidant activity, determination of the proximate composition and nutritional value, study of antimicrobial activity on nine strains of microorganisms and toxicity studies on several cell lines. The present study has a few limitations that provide an opportunity for future research. Thus far, our study focused on obtaining antimicrobial and toxicological data after an initial in vitro biolosgical assessment. Nevertheless, the studies can be continued by testing our two new products on other microbial strains and cell lines. For the sensory analysis the number of panelists used was limited due to the pandemic condition. Thus, to evaluate the consumer’s acceptability regarding the consumption of the two products, a more extensive panel of sensory evaluators can be used. The anticariogenic activity of the products was evaluated only by in vitro studies. Clearly, further clinical tests are needed to reveal the anticariogenic action of these products and to emphasize even more the importance of replacing the classic hot chocolate with sugar, so loved especially by children, with these new products, tasty and with many health benefits.

## 4. Conclusions

Used since ancient times, cocoa-based products continue to represent the option of choice for many people; however, the increased degree of awareness in terms of negative aspects of sugar consumption has created the need for artificial sweeteners with low calories and similar taste. Although originally used, chemical sweeteners have been involved in several health issues thus triggering the search for vegetal sweeteners which ideally would contribute to oral hygiene. The current study proposes the preparation of two hot chocolate products with vegetal compositions which, in addition to agreeable taste, may provide antioxidant and antimicrobial properties. A variety of plant products was used, either herbs of fruits, with high polyphenolic contents and strong antioxidant activity. Both products exhibited antimicrobial effects, in particular on germs that populate the oral cavity; however, the VHC1 product was revealed with the highest antibacterial properties. When the two final products were tested in vitro on human gingival keratinocytes, all tested concentrations not only proved non-toxic but induced a slight stimulatory effect on cell viability thus revealing that both products can be safely used in oral administration. Also, this result may suggest the potential beneficial effects on the oral mucosa thus providing an opportunity for future research. When applied on dermal cell lines, the two chocolate products exhibited safe toxicological profiles even in high concentrations; nevertheless, low concentrations should be used in skin treatments.

In terms of sensory qualities, the results indicated the consumers’ preference for the chocolate based on dehydrated fruit powder (VHC2) which obtained the highest score, being classified as highly acceptable.

Overall, the current work emphasizes the potential of vegetal mixtures to be used as nutraceuticals as well as topic application in skin care because of antimicrobial and antioxidant effects. In addition, their lack of toxicity recommends them as sugar-free potential substitutes for conventional products that can be used as adjuvant and complementary treatment in a healthy diet.

## Figures and Tables

**Table 1:** Recipes for hot chocolate powder variants: vegan hot chocolate 1 (VHC1) and vegan hot chocolate 2 (VHC2).

Common Base Components (CBC)	Cocoa Powder with Max 12% Fat	Carob Powder	Stevia Leaf Powder	Coconut Milk Powder	Soy Lecithin Powder	Vanilla Pods Powder
Commercial brand	“Solaris”	“Solaris”	“Dragon Superfoods”	“Vegis”	“Ecomil”	“Biovegan”
% *	38	11.5	6.5	38	1.9	0.1
VHC1 specific components	Green tea leaves	Alfalfa leaf and steams	Wild thyme aerial parts with flowers	Sage leaves	Echinacea aerial part with flowers	Cardamom pods
Commercial brand	“Sonnentor”	“Phyto Biocare”	“Fares”	“Sonnentor”	“Fares”	“Carmita Classic”
% *	1	1	0.5	0.5	0.5	0.5
VHC2 specific components	Black currant fruit	Blueberry fruit	Hibiscus flowers	Cloves buds		
Commercial brand	“Obio”	“Lyolife”	“Dacia Plant”	“Solaris”		
% *	1	1	1	1		

* from total mass of powdered final product.

**Table 2:** Total polyphenols in raw materials.

	Sample	Total Polyphenols (mg GAE/g)
The basis for both VHC1 and VHC2 recipes	Cocoa powder	109.70 ± 1.93
	Carob powder	102.78 ± 1.79
	Coconut milk powder	17.98 ± 0.90
	Stevia rebaudiana leaf powder	103.36 ± 2.74
	Soy lecithin powder	11.04 ± 0.45
	Vanilla pods powder	101.64 ± 1.69
Specific components in VHC1 recipe	Cardamom powder	44.72 ± 1.33
	Wild thyme powder	108.49 ± 1.88
	Green tea powder	104.89 ± 3.22
	Alfalfa powder	17.14 ± 0.41
	Sage powder	59.14 ± 1.47
	Echinacea powder	21.07 ± 0.54
Specific components in VHC2 recipe	Black currant powder	40.19 ± 0.76
	Blueberry powder	47.09 ± 1.52
	Hibiscus powder	25.78 ± 1.10
	Cloves powder	130.19 ± 2.71
Final products	VHC1 powder	127.81 ± 2.33
	VHC2 powder	137.70 ± 2.23
	VHC1 beverage	17.14 ± 0.55
	VHC2 beverage	18.50 ± 0.52

**Table 3:** Individual polyphenols in raw materials.

	Polyphenol Concentration (mg/g)															
Polyphenol	Cocoa	Coconut Milk	Carob	Stevia Rebaudiana	Cardamom	Vanilla	Thyme	Green Tea	Lecithin	Black Currant	Alfalfa	Blueberry	Sage	Clove	Hibiscus	Echinacea
Gallic acid (GA)	0.79 ± 0.028	-	23.98 ± 0.764	1.52 ± 0.071	0.22 ± 0.007	-	-	1.02 ± 0.028	-	0.14 ± 0.007	0.42 ± 0.014	-	-	3.80 ± 0.184	-	-
Protocatechuic acid (PR)	18.21 ± 0.467	-	23.06 ± 0.580	-	0.39 ± 0.014	3.36 ± 0.141	-	-	-	1.12 ± 0.042	-	-	-	6.21 ± 0.240	-	-
Caffeic acid (CA)	1.63 ± 0.085	0.03 ± 0.007	1.66 ± 0.057	1.39 ± 0.042	0.18 ± 0.014	2.37 ± 0.113	0.36 ± 0.028	24.88 ± 0.566	0.04 ± 0.003	0.49 ± 0.028	0.25 ± 0.014	4.84 ± 0.141	0.73 ± 0.028	3.83 ± 0.085	0.26 ± 0.007	0.15 ± 0.007
Epicatechin (EC)	1.33 ± 0.042	0.05 ± 0.001	29.40 ± 0.933	0.17 ± 0.007	-	35.57 ± 0.594	10.01 ± 0.368	13.05 ± 0.424	-	2.43 ± 0.071	8.55 ± 0.283	6.66 ± 0.255	3.85 ± 0.184	18.33 ± 0.495	8.75 ± 0.283	18.27 ± 0.368
p-Coumaric acid (CU)	-	-	0.50 ± 0.028	0.47 ± 0.014	0.34 ± 0.014	4.65 ± 0.099	0.75 ± 0.042	3.04 ± 0.099	-	0.06 ± 0.004	0.15 ± 0.004	0.07 ± 0.003	0.05 ± 0.001	0.74 ± 0.028	0.13 ± 0.007	0.08 ± 0.004
Ferulic acid (FE)	0.82 ± 0.028	-	0.30 ± 0.014	0.29 ± 0.007	0.65 ± 0.014	0.20 ± 0.001	0.44 ± 0.014	2.20 ± 0.071	-	0.08 ± 0.002	0.06 ± 0.002	0.04 ± 0.001	0.10 ± 0.002	0.28 ± 0.014	-	-
Rutin (RU)	0.05 ± 0.001	-	1.55 ± 0.071	-	-	4.15 ± 0.071	3.32 ± 0.113	8.41 ± 0.226	-	0.47 ± 0.028	0.48 ± 0.028	1.86 ± 0.057	1.99 ± 0.057	16.13 ± 0.325	1.96 ± 0.057	1.13 ± 0.04
Rosmarinic acid (RO)	-	0.05 ± 0.001	6.35 ± 0.141	0.86 ± 0.028	-	2.22 ± 0.057	0.09 ± 0.003	0.25 ± 0.014	-	0.46 ± 0.021	0.19 ± 0.014	0.22 ± 0.006	0.79 ± 0.028	4.34 ± 0.099	0.51 ± 0.014	0.61 ± 0.028
Resveratrol (RS)	-	-	0.86 ± 0.0028	1.94 ± 0.056	-	1.90 ± 0.07	6.06 ± 0.198	0.08 ± 0.002	-	0.03 ± 0.001	0.11 ± 0.004	0.22 ± 0.007	16.08 ± 0.467	4.54 ± 0.099	0.20 ± 0.007	5.35 ± 0.141
Quercitin (QU)	2.47 ± 0.099	0.01 ± 0.001	1.90 ± 0.071	4.87 ± 0.099	0.01 ± 0.001	2.52 ± 0.099	2.34 ± 0.057	0.15 ± 0.007	-	0.02 ± 0.001	0.14 ± 0.004	0.04 ± 0.001	2.04 ± 0.042	3.50 ± 0.071	0.11 ± 0.003	2.05 ± 0.057
Kaempherol (KP)	0.56 ± 0.028	0.07 ± 0.003	1.70 ± 0.071	4.36 ± 0.085	0.06 ± 0.002	3.72 ± 0.099	4.57 ± 0.141	0.04 ± 0.001	0.04 ± 0.001	0.31 ± 0.007	0.31 ± 0.011	0.05 ± 0.003	1.25 ± 0.057	14.45 ± 0.566	0.86 ± 0.028	2.10 ± 0.071

“-”: no traces of individual polyphenols were found.

**Table 4:** Antioxidant activity (mg Trolox/g) of final products.

Parameters	VHC1 Beverage	VHC2 Beverage
Antioxidant activity (mg Trolox/g)	38.608 ± 1.609	37.258 ± 1.826

Each value was the mean of duplicate measurements. Values are expressed as mean ± SD.

**Table 5:** Proximate composition of final products.

Parameters	VHC1 Powder	VHC2 Powder
Moisture (g/100 g)	4.08 ± 0.1	3.85 ± 0.05 *
Mineral substances (g/100 g)	5.86 ± 0.174	5.03 ± 0.101 *
Proteins (g/100 g)	11.11 ± 0.245	11.45 ± 0.390
Lipids (g/100 g):	9.56 ± 0.181	9.04 ± 0.165
- saturated fatty acids (g/100 g)	6.42 ± 0.121	6.13 ± 0.106 *
Carbohydrates (g/100 g):	69.36	71.63
- sugar (g/100 g)	15.35 ± 0.176	15.85 ± 0.165 *
- dietary fiber (g/100 g)	28.37 ± 1.068	29.3 ± 1.005
Nutritional value (kcal/100 g)	408.04	404.68

Each value was the mean values and standard deviations of all measurements; * superscript between final products indicate significant difference (*p* < 0.05). Carbohydrates and nutritional value were obtained by calculation.
